# Analysis of necroptosis and its association with pyroptosis in organ damage in experimental pulmonary arterial hypertension

**DOI:** 10.1111/jcmm.17272

**Published:** 2022-04-07

**Authors:** Izabela Jarabicová, Csaba Horváth, Eva Veľasová, Lenka Bies Piváčková, Jana Vetešková, Ján Klimas, Peter Křenek, Adriana Adameová

**Affiliations:** ^1^ 37864 Faculty of Pharmacy Department of Pharmacology and Toxicology Comenius University in Bratislava Bratislava Slovakia

**Keywords:** necroptosis, pulmonary arterial hypertension, pyroptosis, receptor‐interacting protein kinase 3

## Abstract

In this study, a role of cell loss due to necroptosis and its linkage with pyroptosis in organ damage under the conditions of pulmonary arterial hypertension (PAH) was examined. Monocrotaline (MCT) was used to induce PAH in Wistar rats, and depending on the severity of the disease progression, they were further divided into two subgroups: MCT group—sacrificed 4 weeks after MCT administration and ptMCT group—prematurely sacrificed due to rapid deterioration in vital functions (on Day 24,11 ± 0,7). The elevation of respiratory rate and right ventricular (RV) hypertrophy were more evident in ptMCT group, while the heart rate and cardiac haemodynamic stress markers were comparably higher in both diseased groups. Detailed immunoblotting analysis revealed that the upregulation of pThr^231^/Ser^232^‐RIP3 proceeded into necroptosis execution in the RVs, unlike in the lungs of both PAH stages. The elevated pulmonary pThr^231^/Ser^232^‐RIP3 levels in both PAH subgroups were associated rather with GSDMD‐mediated pyroptosis. On the contrary, other inflammasome forms, such as AIM2 and NLRC4, were higher in the RV, unlike in the lungs, of diseased groups. The PAH‐induced increase in the plasma RIP3 levels was more pronounced in ptMCT group, and positively correlated with RV hypertrophy, but not with haemodynamic stress. Taken together, we indicated for the first time that pThr^231^/Ser^232^‐RIP3 upregulation resulting in two different necrosis‐like cell death modes might underlie the pathomechanisms of PAH and that the plasma RIP3 might serve as an additional diagnostic and prognostic marker of cardiac injury under these conditions.

## 1 INTRODUCTION

Over the past two decades, necroptosis, a form of programmed necrosis, has been suggested to underlie the pathogenesis of various cardiovascular diseases and its significant role has been highlighted in myocardial ischaemia‐reperfusion injury^1^, ^2^, ^3^, ^4^ and heart failure.^4^, ^5^, ^6^, ^7^ At the molecular level, the canonical necroptotic signalling is dependent on the assembly of an amyloid‐like protein complex referred to as ‘the necrosome’, which consists of receptor‐interacting protein kinase 1 (RIP1), RIP3 and mixed lineage kinase domain‐like protein (MLKL),^8^ although the requirement of the former protein kinase of RIP family for necroptosis induction has been questioned.^9^, ^10^ As an initiation step towards the activation of necroptosis, RIP3 undergoes auto/RIP1‐mediated phosphorylation^11^ and such post‐translational modifications lead to phosphorylation, oligomerization and subsequent translocation of the terminal executive protein MLKL to the plasma membrane promoting its rupture and eventually cell death.^12^ Besides mediating necroptosis, both RIP3 and MLKL have been indicated to interact with other signalling molecules of inflammatory response via the activation of the NLR family pyrin domain containing 3 (NLRP3) inflammasome.^13^, ^14^, ^15^ Thus, such interaction might represent an important convergence of necroptosis and pyroptosis—another cell damaging process resembling necrotic phenotype.^16^

Pulmonary arterial hypertension (PAH) refers to a progressive and multifactorial disorder characterized by extensive damage of the pulmonary vasculature and the development of right ventricular (RV) heart failure, the leading cause of death in these patients.^17^ The main pathomechanisms being causal for PAH progression include vasoconstriction, proliferative changes and remodelling of pulmonary vessels.^18^ The currently used therapeutic interventions for the treatment of PAH are only of modest efficacy and, most importantly, do not decrease mortality.^19^ Thus, it is very likely that the molecular events standing behind these functional and morphological alterations of PAH are not sufficiently targeted. In this regard, programmed cell death accompanied by inflammatory response seems to be a subject of interest. Indeed, the alterations in mRNA levels of some necroptotic signalling molecules have been found in monocrotaline (MCT)‐induced PAH.^20^ Likewise, in Sugen/hypoxia model of PAH, it has been indicated that necroptosis might be responsible for the increase of circulating levels of high mobility group box 1 protein (HMGB1), the well‐recognized mediator of inflammation and vascular repair in PAH.^21^

Based on these two literary indications,^20^, ^21^ and by following the guidelines for evaluating myocardial cell death,^22^ we aimed to provide a complex analysis of necroptosis in both lungs and right ventricles (RVs) of rats with MCT‐induced PAH. Furthermore, since necroptosis can act as an upstream activator of pyroptosis,^23^ which in turn has also been shown to participate in the pathomechanisms of PAH,^24^, ^25^ we examined some critical pyroptotic markers as well, and thereby delineated the potential interconnection of these two cell death forms under such pathological circumstances. The comprehensive molecular analyses of these particular necrosis‐like cell death programmes were performed in two different stages of MCT‐induced PAH, namely 4 weeks after MCT administration, and in the more advanced stage of PAH characterized by remarkable signs of rapid deterioration in health. Moreover, we examined the plasma levels of RIP3 in the diseased animals, and thereby elucidated whether the levels of this circulating protein kinase could be associated with the disease severity, and thus could serve as a potential diagnostic and prognostic marker of cardiac injury under PAH conditions.

## 2 MATERIALS AND METHODS

### 2.1 Animals and experimental design

Male Wistar rats (10–12 weeks old) were used (Department of Toxicology and Laboratory Animals breeding, Slovak Academy of Sciences, Dobra Voda, Slovak Republic). The animals were kept under standard laboratory conditions with access to food and water ad libitum. All procedures using experimental animals were approved by the Ethical Committee of the Faculty of Pharmacy and by the State Veterinary and Food Administration of the Slovak Republic (Ro‐108/18‐221/3). The investigation conforms to the Guide for the Care and Use of Laboratory Animals: Eight Edition (2010) published by the US Committee for the Update of the Guide for the Care and Use of Laboratory Animals; National Research Council, and with the EU adopted Directive 2010/63/EU of the European Parliament and of the Council on the protection of animals used for experimental and other scientific purposes and to the Slovak law regulating animal experiments.

**FIGURE 1:**
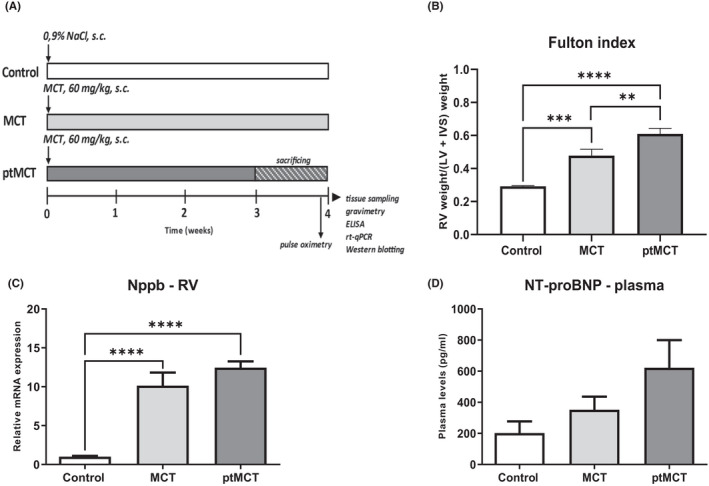
Schematic illustration of experimental protocol (A), marker of cardiac hypertrophic injury (B) and haemodynamic stress markers in the RV tissue and plasma (C–D). Control—control group administrated vehicle and sacrificed after 28 days; MCT—group administrated monocrotaline to induce PAH and sacrificed after 28 days; ptMCT—group administrated monocrotaline to induce PAH and prematurely sacrificed due to rapid progression of the disease and health deterioration on average on day 24,11 ± 0,7. Data are presented as mean ± SEM; *n* = 6–10 per group; * *p* ˂ 0.05

Schematic design of this study is depicted in Figure 1A. Rats were administered monocrotaline (MCT, 60 mg/kg, s.c.) to induce PAH while control animals received vehicle only (0.9% NaCl, s.c.). All rats were weighed weekly until week 3. From Day 21, the rats health visual monitoring and weighting was provided twice a day for detailed inspection of health status. Animals showing the signs of health deterioration as a part of rapid progression of PAH, such as loss of body weight of more than 10 g in 12 h, bristled fur, apathy, low temperature of acral body parts or dyspnoea were considered to have progressed to more advanced stage and were prematurely (on Day 24,11 ± 0,7 on average) sacrificed to humanely end the experiment (ptMCT group). Two independent examiners judged these health conditions. The MCT‐treated animals that did not show acute deterioration were kept until Day 28 when they were sacrificed (MCT group).

Heart rate, haemoglobin oxygen saturation and breathing frequency were measured by pulse oximeter 24 h before sacrifice in MCT group and shortly before sacrifice in ptMCT group. Sensor collars of appropriate size were placed around the neck of rats in conscious animals according to the instructions of manufacturer. The vital functions were recorded and analysed on calm, still animals and on moving animals under passivity and activity conditions, respectively (MouseOx Plus, Starr Life Sciences,).

Rats were sacrificed by CO_2_ asphyxiation, EDTA plasma was prepared, and tissue samples were collected and stored at −80°C until further analysis (Figure 1A).

### 2.2 Reverse transcription‐quantitative polymerase chain reaction (RT‐qPCR)

RT‐qPCR was performed on total RNA samples of lungs and RVs, as described previously.^26^, ^27^ The resulting gene expressions of brain natriuretic peptide (Nppb), Nlrp3 and NLR family CARD domain containing 4 (Nlrc4) were calculated according to Pfaffl (2001)^28^ and normalized to endogenous reference gene hypoxanthine‐guanine phosphoribosyltransferase (Hprt1), whose expression is considered to be constitutive in a given tissue. Finally, the expression of the target genes was calibrated for the expression in control group. The sequences of primers are presented in (Table S1). All results are expressed relative to the average of control group.

### 2.3 ELISA

For N‐terminal pro‐brain natriuretic peptide (NT‐proBNP) and RIP3 detection, EDTA plasma was prepared from venous blood following sacrifice. Aliquots of the plasma were stored at −80°C until further processing. We used rat NT‐proBNP ELISA Kit (SKU E‐EL‐R0670, ElabSiences) and rat RIP3 ELISA Kit (LS‐F35895‐1, LSBio,) according to the manufacturer's instructions.

### 2.4 Western blotting

SDS‐PAGE and immunoblotting were performed as described previously.^29^ Briefly, lung and RV tissue samples were used for semiquantitative protein expression analysis. Prepared samples (30 μg/lane) were electrophoresed using SDS‐PAGE under reducing conditions (Bis‐tris/MOPS 10% gels,) and transferred to polyvinylidene difluoride membranes (Immobilon‐P^®^, Merck Millipore). Membranes were blocked for 30 min at room temperature and incubated with the following primary antibodies: RIP3 (#15828, Cell Signaling Technology), pThr^231^/Ser^232^‐RIP3 (ab222320, Abcam), MLKL (ab243142, Abcam), pSer^345^‐MLKL (MABC‐1158, Merck), caspase‐8 (#4790 Cell Signaling Technology), caspase‐1 (ab179515, Abcam), IL‐1β (ab9722, Abcam), GSDMD (sc‐81868), TNF (ab66579, Abcam), HMGB1 (ab18256, Abcam), AIM2 (ab119791, Abcam) and Iba1 (ab108539, Abcam). These were used in conjunction with appropriate secondary antibodies: donkey anti‐rabbit [H+L] IgG‐HRP (711–035–152, Jackson Immunoresearch), goat anti‐mouse [L] IgG‐HRP (115–035–174, Jackson Immunoresearch) and donkey anti‐rat IgG‐HRP (112–035–175, Jackson Immunoresearch). Signals generated using an enhanced chemiluminescence (Crescendo Luminata, Merck Millipore) were captured by a chemiluminescence imager (myECL Imager, Thermo Scientific) and analysed with myECL Image Analysis software (Thermo Scientific). Final relative intensities corresponding to protein expression were calculated as a ratio of a target epitope signal to the total protein staining (whole‐lane post‐transfer protein staining with Ponceau S) in its lane.^30^

### 2.5 Statistical analysis

Data are presented as mean ± standard error of the mean. To compare groups, we used one‐way ANOVA followed by the Tukey's test or the Kruskal–Wallis test followed by the Dunn's test. For the correlation analyses, Pearson correlation was applied. Differences with *p* < 0.05 were considered to be significant. All analyses were performed by using GraphPad Prism 8.0.1 (GraphPad Software, Inc.,).

## 3 RESULTS

### 3.1 Gravimetry

Due to MCT treatment, body weight of the rats decreased, and such decrease was more evident in ptMCT group, indicating relatively rapid progression of the disease. The absolute weight of the isolated RV, the weight of RV after normalization to body weight and Fulton index [calculated as RV/(LV+S)] were significantly increased in both PAH groups with a greater RV mass and RV hypertrophy in the progressive stage of the disease (Table S2, Figure 1B).

### 3.2 Influence of monocrotaline on animal vital functions

Analyses of some vital signs under conditions of passivity and activity are shown in Table S3. There were no changes in haemoglobin oxygen saturation among groups. Heart rate was increased due to MCT treatment and did not differ between the two PAH groups. In contrast, respiratory rate was significantly increased in ptMCT group compared with both MCT and control group. These data are compared with the results of vital signs in active animals (Table S3).

### 3.3 Relative mRNA expression of Nppb in the RV and plasma NT‐proBNP concentration

Nppb mRNA expression was significantly increased in the RV of the diseased groups while the significance did not change with the higher severity of PAH (Figure 1C). However, at the protein level, there were no significant changes in the plasma NT‐proBNP concentrations among the diseased and non‐diseased groups (Figure 1D).

### 3.4 Necroptotic canonical pathway

**FIGURE 2:**
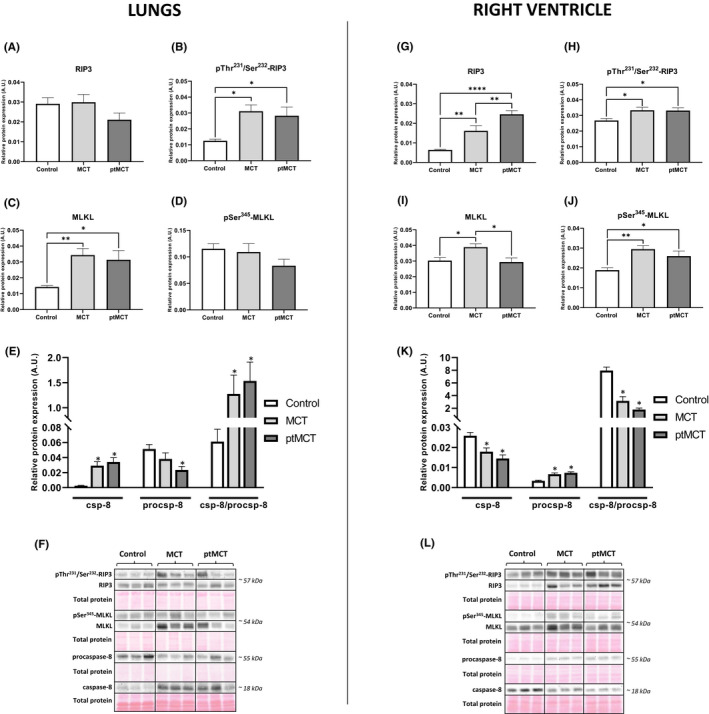
Analysis of necroptotic signalling in the lung (left panel) and RV (right panel) tissue. Relative protein expression (A–E, G–K) and representative immunoblots and total protein staining (F, L) of RIP3 (A, G), pThr^231^/Ser^232^‐RIP3 (B, H), MLKL (C, I), pSer^345^‐MLKL (D, J), csp‐8 and procsp‐8 (E, K) in control group, monocrotaline group (MCT) and prematurely terminated monocrotaline group (ptMCT). Data are presented as mean ± SEM; *n* = 9–10 per group; * *p* ˂ 0.05

Protein analysis of the main necroptotic mediators in PAH‐affected lung and RV tissue is shown in Figure 2A–F and Figure 2G–L (left and right side, respectively). While the expression of total RIP3 in the lungs did not differ among groups (Figure 2A), its phosphorylated form, pThr^231^/pSer^232^‐RIP3, was significantly increased in both PAH groups irrespectively of the disease severity (Figure 2B). MCT treatment comparably elevated the levels of total MLKL in both stages of PAH (Figure 2C); however, the analysis of its phosphorylated form, pSer^345^‐MLKL, which is strongly associated with necroptosis execution,^22^ revealed no differences among the control and diseased groups (Figure 2D). The levels of caspase‐8 (csp‐8), known to act as a negative regulator of this cell death mode by cleaving RIP kinases,^31^ were significantly elevated in the MCT‐treated groups and csp‐8/procsp‐8 ratio mimicked this pattern of results (Figure 2E).

In the RVs of both stages of PAH, the levels of RIP3 were significantly increased with being higher in ptMCT group (Figure 2G). Consistently, pThr^231^/pSer^232^‐RIP3 was equally upregulated in both stages of the disease (Figure 2H). The expression of total MLKL was significantly elevated in MCT group; however, in the progressive stage of PAH, the levels of this protein were compared with those of control group (Figure 2I). Its phosphorylated form, pSer^345^‐MLKL, was equally upregulated in both PAH stages (Figure 2J). The active fragment of csp‐8 was significantly reduced in both MCT groups, while its inactive form, procsp‐8, was increased due to MCT treatment (Figure 2K).

Based on these data showing the likely execution of necroptosis in the RV of the rats with PAH, we further investigated correlations between the necroptotic markers and some other well‐established markers of cardiac injury. Pearson correlation analysis revealed that the RV levels of RIP3 positively correlated with the mRNA levels of Nppb (*p* < 0.05; *r* = 0.4828), the plasma levels of NT‐proBNP (*p* < 0.05; *r* = 0.6267) and Fulton index (*p* < 0.05; *r* = 0.5618) (Figure S1A–C). On the contrary, no correlation was found by investigating the expression of pThr^231^/pSer^232^‐RIP3 and pSer^345^‐MLKL with respect to the levels of Nppb, NT‐proBNP and Fulton index (Figure S1D‐I).

**FIGURE 3:**
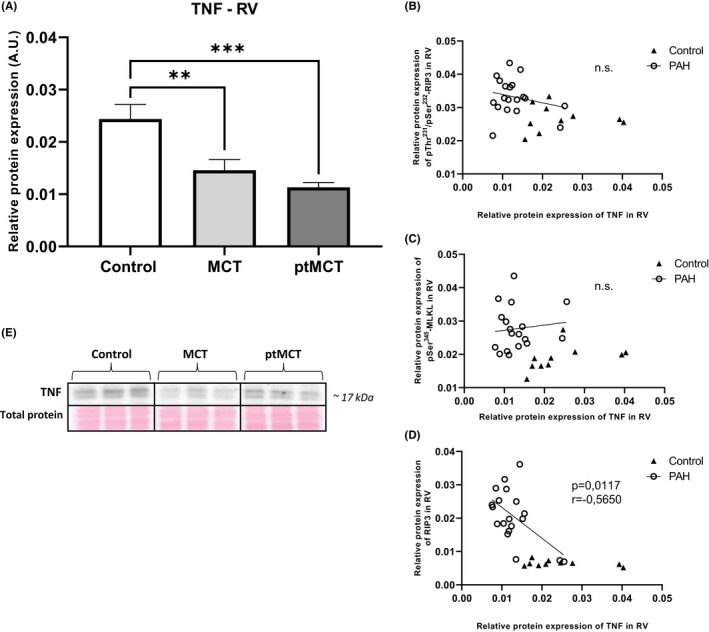
Analysis of TNF expression and its correlation with the main necroptotic markers in the RV tissue. Relative protein expression (A) and representative immunoblots and total protein staining (E) of TNF in control group, monocrotaline group (MCT) and prematurely terminated monocrotaline group (ptMCT). Correlation between TNF and pThr^231^/Ser^232^‐RIP3 (B), pSer^345^‐MLKL (C), RIP3 (D) in monocrotaline group (MCT) and prematurely terminated monocrotaline group (ptMCT). Data are presented as mean ± SEM; *n* = 9–10 per group; * *p* ˂ 0.05; n.s.—non‐significant

In addition to searching for a link between the markers of cardiac injury and necroptotic markers, we also examined a possible association between necroptosis activation and tumour necrosis factor (TNF) levels. Interestingly, the levels of TNF were decreased in the RV of both PAH groups (Figure 3A,E), and the correlation analysis revealed that there is unlikely a link between neither TNF and pThr^231^/pSer^232^‐RIP3 nor TNF and pSer^345^‐MLKL RV levels (Figure 3B,C). On the contrary, negative correlation between TNF and RIP3 expression in the RV was found (*p* < 0.05; *r* = −0.5650) (Figure 3D).

### 3.5 Plasma concentration of RIP3

**FIGURE 4:**
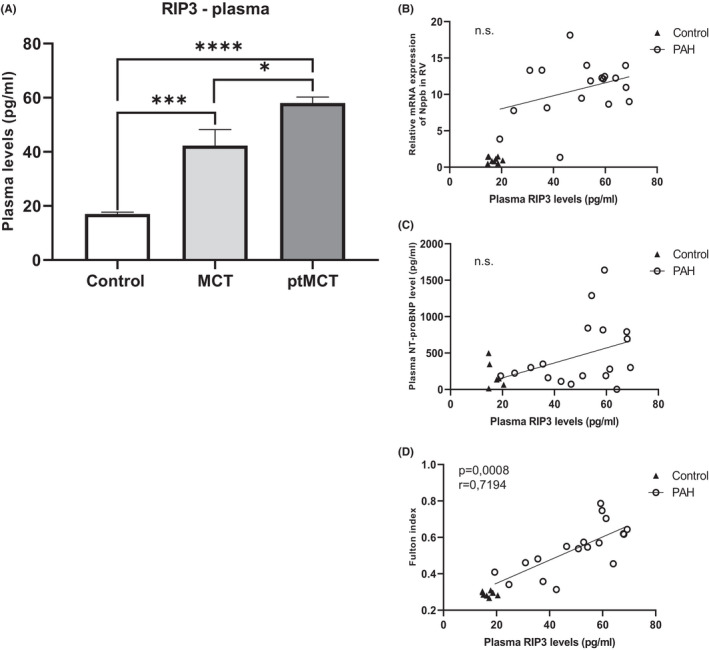
Analysis of the plasma levels of RIP3 and their correlation with markers of cardiac injury. The plasma levels of RIP3 (A) in control group, monocrotaline group (MCT) and prematurely terminated monocrotaline group (ptMCT). Correlation between the plasma RIP3 and Nppb in the RV (B), the plasma NT‐proBNP (C), Fulton index (D) in monocrotaline group (MCT) and prematurely terminated monocrotaline group (ptMCT). Data are presented as mean ± SEM; *n* = 9 per group; *p ˂ 0.05; n.s.—non‐significant

The plasma RIP3 concentrations, of which higher levels were found in the conditions of compromised heart function^32^, ^33^, were evaluated by using ELISA assay. PAH induction caused significant increase in the plasma RIP3 levels with more remarkable effects in the more advanced stage of the disease (Figure 4A).

To examine whether the changes in the plasma RIP3 levels are associated with some clinical signs of the disease, we next performed correlation analyses. Despite the plasma RIP3 being increased due to PAH, no impact on either heart or breath rate under passivity or activity conditions was found (Figure S2A–D). Likewise, we did not find any correlation between the plasma RIP3 levels and other markers of cardiac damage, such as the mRNA levels of Nppb in the RV and the plasma NT‐proBNP levels (Figure 4B,C). In contrast, the plasma RIP3 was positively correlated with Fulton index (*p* < 0.05; *r* = 0.7194) (Figure 4D).

### 3.6 Pyroptotic pathway

**FIGURE 5:**
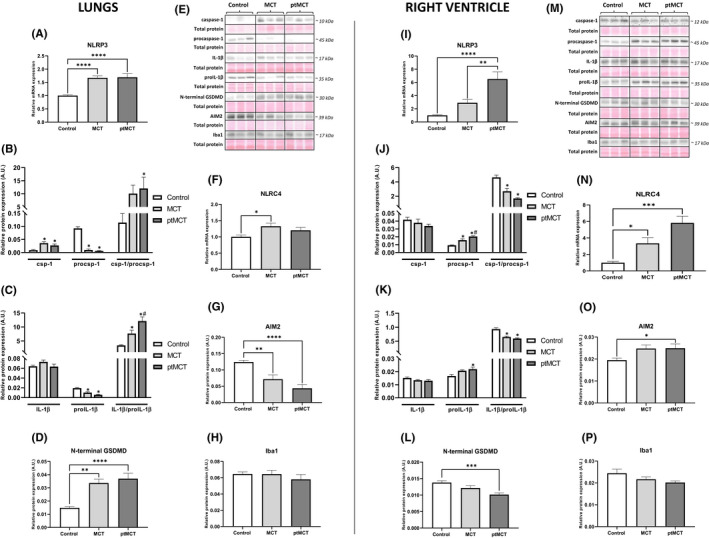
Analysis of inflammasomes and pyroptotic signaling in the lung (left panel) and RV (right panel) tissue. Relative mRNA expression of NLRP3 (A, I) and NLRC4 (F, N), relative protein expression (B‐D, G, H, J–L, O, P) and representative immunoblots and total protein staining (E, M) of csp‐1, procsp‐1 (B, J), IL‐1β, pro‐IL‐1β (C, K), N‐terminal GSDMD (D, L), AIM2 (G, O) and Iba1 (H, P) in control group, monocrotaline group (MCT) and prematurely terminated monocrotaline group (ptMCT). Data are presented as mean ± SEM; *n* = 8–10 per group; *p ˂ 0.05 vs. Control; ^#^ *p* ˂ 0.05 vs. MCT

Because pro‐necroptotic proteins RIP3 and MLKL, in particular their unphosphorylated forms, have also been shown to modulate NLRP3 inflammasome complex formation and thereby promoting pyroptosis,^13^, ^14^, ^15^ the expression of key pyroptotic markers in the lung and RV tissue was also investigated (Figure 5A–H and 5I–P respectively). The levels of NLRP3 in both tissues were assessed by RT‐qPCR since we were not able to detect a signal for this protein by immunoblotting, even though various antibodies purchased from different manufacturers were used. The mRNA expression of NLRP3 was found to be upregulated in both the lung and RV tissue due to PAH (Figure 5A,I). In the lungs, cleavage of caspase‐1 (csp‐1), which plays a role in the canonical pathway of pyroptosis,^23^ was increased in both diseased groups, while MCT group was close to reaching statistical significance (*p* = 0.067) (Figure 5B). In line with these results, MCT treatment increased the relative processing of interleukin‐1β (IL‐1β) which was more pronounced in the progressive stage of PAH (Figure 5C). The levels of N‐terminal gasdermin D (GSDMD), which forms pores in the plasma membrane and thereby mediates pyroptosis execution,^32^ were significantly higher in both MCT groups than in control group, and no changes between the two PAH stages were observed (Figure 5D).

Contrary to the results in the lung tissue, there was a significant decrease in csp‐1 cleavage in the RV of both PAH groups (Figure 5J). The levels of IL‐1β did not differ among groups, while its precursor pro‐IL‐1β was significantly elevated in ptMCT group and close to statistical significance in MCT group (*p* = 0.0634) (Figure 5K). Interestingly, a pore‐forming N‐terminal fragment of GSDMD was decreased in RV of both stages of PAH (Figure 5L).

In addition to NLRP3, other main inflammasome sensors, such as NLRC4 and absent in melanoma 2 (AIM2), known to be upregulated in circumstances associated with cardiac injury,^33^, ^34^ were also investigated. The expression of NLRC4 was assessed by RT‐qPCR due to similar technical issues as in the case of NLRP3 evaluation. The mRNA levels of NLRC4 in the lung tissue were elevated in PAH groups, while reaching the significance in MCT group only. In the RV, the mRNA levels of NLRC4 were upregulated in both stages of the disease (Figure 5F,N). The AIM2 levels of the RV were significantly increased and close to statistical significance (*p* = 0.0631) in ptMCT and MCT group respectively (Figure 5O). In the lungs, downregulation of this inflammasome sensor was even found (Figure 5G). Because inflammasomes are predominantly expressed by the innate immune system,^35^ their source in the RV and lungs was evaluated by identification of ionized calcium‐binding adapter molecule 1 (Iba1), a specific marker of monocytes/macrophages.^36^ Interestingly, the expression of Iba1 did not differ among the groups in either of the investigated tissue (Figure 5H,P).

### 3.7 HMGB1

**FIGURE 6:**
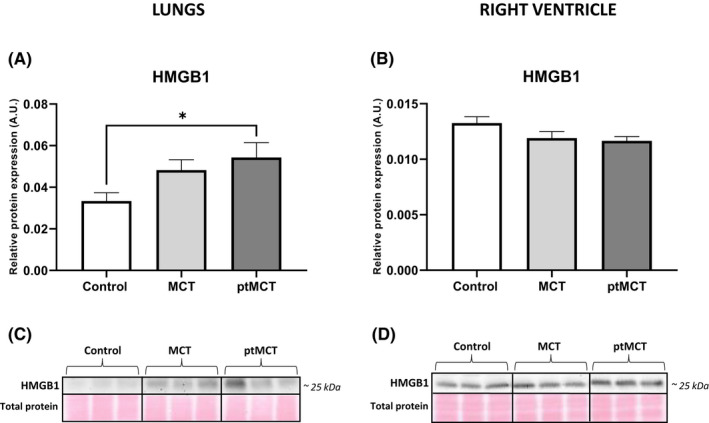
Analysis of HMGB1 expression in the lung and RV tissue. Relative protein expression (A, B) and representative immunoblots and total protein staining (C, D) of HMGB1 in control group, monocrotaline group (MCT) and prematurely terminated monocrotaline group (ptMCT). Data are presented as mean ± SEM; *n* = 9–10 per group; *p ˂ 0.05

Because HMGB1 has been recognized as a nonspecific marker indicating the plasma membrane rupture and a mediator of inflammation,^37^ we also examined its levels. In the lungs, HMGB1 levels were higher due to MCT treatment, although the significant changes were found in the more advanced stage only (Figure 6A,C). In the RV, no changes in the expression of HMGB1 among groups were observed (Figure 6B,D).

## 4 DISCUSSION

This is the first study providing a complex protein analysis of necroptosis signalling and its interconnection with pyroptosis in the lungs and RVs of MCT‐treated rats experiencing two differently severe stages of PAH, and thereby bringing novel insights into the molecular mechanisms of this disease. Our results showed that the investigated programmed necrosis‐like cell death modalities might mediate, at least in part, cellular damage in these two PAH‐affected tissues. Namely, we reported that necroptosis likely contributes to RV injury due to PAH, as evidenced by the increased levels of both pThr^231^/Ser^232^‐RIP3 and pSer^345^‐MLKL. On the contrary, increased phosphorylation of RIP3 in the lungs did not result in the activation of MLKL and the pyroptotic csp‐1–IL‐1β–GSDMD molecular axis was rather responsible for the damage of this tissue. In addition, the animals of both PAH stages exhibited elevated plasma levels of RIP3 while being significantly higher in the more progressive stage suggesting its potential as a more sensitive biomarker when compared with non‐significant sensitivity of NT‐proBNP. Moreover, the plasma RIP3 levels were positively correlated with RV hypertrophy, but not with the markers of haemodynamic stress. Thus, these findings may indicate a complex role of RIP3 in multi‐organ damage due to PAH and highlight the screening of the plasma RIP3 as an additional diagnostic and prognostic marker of cardiac injury.

Recently, it has been shown that mRNA levels of both RIP3 and MLKL in the rat lungs with MCT‐induced PAH are increased and thereby necroptosis has been suggested as a potential mechanism mediating pulmonary vascular remodelling and inflammation under such conditions.^20^ However, according to the current guidelines for evaluating myocardial cell death modalities,^22^ such transcriptomic analysis does not provide robust evidence on necroptosis and therefore, more specific markers of this cell death should be assessed, instead. Thus, consistently with these recommendations, in this study, both protein expression and changes in phosphorylation state of key necroptosis mediators, which are considered to underlie the executive mechanisms of a particular cell death programme, were evaluated for the first time. Our findings showed that the total expression of RIP3 was unchanged in the lungs of both stages of PAH; however, its active phosphorylated form pThr^231^/Ser^232^‐RIP3, serving as an upstream molecule of MLKL,^8^ was elevated in such diseased tissue. On the contrary, the pulmonary levels of pSer^345^‐MLKL, which is known to form homo‐/hetero‐oligomers mediating the plasma membrane rupture due to necroptosis,^12^, ^38^, ^39^, ^40^ were unchanged in PAH groups, despite its total form being upregulated in both disease stages. These results clearly indicate that pThr^231^/Ser^232^‐RIP3 in PAH‐affected lungs did not proceed to cytotoxic MLKL activation, and thus necroptosis, and in particular its canonical pathway, is unlikely responsible for the lung tissue damage due to PAH. Although potential differences in findings can occur due to various methodological approaches employed in our study and the study of Xiao et al.^20^ (e.g. protein versus transcriptomic analysis), both of these studies consistently report the increase in RIP3 and MLKL in the rat lungs with PAH while the conclusions are different. To gain a more complex picture of necroptosis signalling, we also evaluated the levels of csp‐8, a negative marker of this cell death. In fact, it prevents necroptosis via cleavage of both RIP1 and RIP3, but at the same time can promote extrinsic apoptosis.^31^ We observed increased activation of csp‐8 in both stages of PAH which is consistent with our other findings on canonical necroptosis signalling in the lungs. The apoptotic downstream molecules of csp‐8 were not a subject of this study; however, it can be noted that the role of apoptosis in the pulmonary damage due to PAH is still not fully known. On the one hand, it has been suggested that the activation of the apoptotic programme in pulmonary vascular endothelial cells is one of the crucial factors contributing to the development of PAH.^41^, ^42^ On the other hand, resistance to apoptosis in both pulmonary vascular endothelial and smooth muscle cells has also been reported, and indeed it has been viewed as a promoter of hyperproliferative changes in pulmonary vasculature in PAH.^43^, ^44^

Besides apoptosis and necrosis‐like cell death modes examined in this study, the potential implication of other cell death programmes in the pathogenesis of PAH could also be taken into account. In fact, based on the screening of ferroptosis‐associated genes and the construction of gene‐microRNA and gene‐transcription factor networks, ferroptotic cell death has recently been suggested as a potential pathomechanism promoting the development of PAH.^45^ Moreover, as it is known that ferroptosis is strongly associated with the accumulation of oxidized components of cell membranes^46^ and overall oxidative stress,^47^ which in turn has been suggested to promote necroptosis signaling,^48^ such interlink between these two cell death forms may also support the hypothesis on a critical role of ferroptotic cell death in PAH.

To the best of our knowledge, necroptosis in the context of RV injury in PAH has not been studied yet, and this is the first study showing necroptotic cell death being activated in such tissue. We found upregulated pThr^231^/Ser^232^‐RIP3 in both PAH stages and its total form, which was, however, more pronounced in the more advanced stage. The expression of the pore‐forming necroptotic pSer^345^‐MLKL was elevated in both stages of PAH and the levels of total MLKL mimicked this pattern in the earlier PAH stage, but its level in the progressive stage was similar to that of control group. In line, the activation of csp‐8 was found to be decreased in both PAH groups, thereby indicating the promotion of necroptosis. All these findings on necroptosis signalling in the RV affected by PAH are novel and very valuable. In fact, necroptosis has been shown to mediate myocardial damage in various heart diseases, such as ischaemia‐reperfusion injury, myocardial infarction and heart failure,^1^, ^2^, ^3^, ^4^, ^5^, ^6^, ^7^ but under the conditions of PAH, it has not been previously studied. Although it could be hypothesized that the more serious stage of PAH, the more pronounced necroptosis in the RV, we have not fully proven this assumption since an equal increase in pThr^231^/Ser^232^‐RIP3 and pSer^345^‐MLKL in both stages of the disease was found. We have no clear clarification for such observations, and we could hypothesize that in the more advanced stage, the heteromers RIP3–MLKL instead of MLKL homomers as a consequence of previous phosphorylations can predominate^49^ and thereby underlie necroptosis. On the contrary, in support of our findings, we found no correlation between the necroptotic markers pThr^231^/Ser^232^‐RIP3 and pSer^345^‐MLKL and RV mRNA levels of Nppb, a gene encoding NT‐proBNP which serves as a clinical marker of myocardial injury due to haemodynamic stress.^50^ Similar results were observed in the case of correlation analysis between these markers of necroptosis and the plasma NT‐proBNP levels. With respect to this, it should be, however, mentioned that a great variability of the data on the plasma NT‐proBNP likely caused that statistical significance was not reached. In contrast, a positive correlation between RV expression of total RIP3 and both Nppb and NT‐proBNP was found, highlighting a prominent role of RV RIP3 in haemodynamic stress due to PAH. Of note, in this study, we also showed a positive correlation between the RV levels of RIP3 and RV hypertrophy. This observation is in line with studies where RIP3 has been found to mediate myocardial remodelling in both necroptosis‐dependent and independent manner.^4^, ^51^ Based on our original findings highlighting RIP3 in the RV, and previous studies showing the higher circulating levels of RIP3 in some cardiac diseases,^52^, ^53^, ^54^ we further investigated its potential clinical relevance in PAH. We delineated that the plasma RIP3 levels are increased due to PAH being more evident in the advanced stage. Moreover, the plasma RIP3 levels positively correlated with Fulton index, but not with vital functions or mRNA levels of Nppb and NT‐proBNP plasma levels. Taken together, it can be suggested that the plasma levels of RIP3 might provide an additional information on cardiac hypertrophy, but not on haemodynamic stress and might serve as a potential diagnostic and prognostic marker of hypertrophic injury due to necroptosis under conditions of PAH.

The signalling of necroptosis induced by TNF ligation on TNFR1 has been the most studied yet.^55^ In our previous study, we have found increased TNF expression along with active necroptosis in post‐infarction heart failure,^5^ and thereby supporting a link between this cytokine and this necrosis‐like cell death programme. From the findings of the present study showing the downregulation of TNF in the RV in both PAH stages, as well as from a study using a model of acute IR,^29^ a similar assumption of pro‐necroptotic action of TNF cannot be fully adopted. In support, we found no correlation between TNF and both pThr^231^/Ser^232^‐RIP3 and pSer^345^‐MLKL and even more, a negative correlation between TNF and RIP3 levels in the RV was documented. Thus, other deleterious stimuli apart from TNF^56^ can be hypothesized to mediate necroptosis in the PAH‐affected RV in this study. In this regard, it can be noted that HMGB1, which is able to activate TLR4 with resultant necroptosis activation,^57^ has been suggested to underlie pulmonary injury due to PAH.^58^, ^59^ This damage‐associated molecular pattern, however, does not seem to be responsible for necroptosis in the diseased RVs of our study, because the RV levels of HMGB1 were unchanged in both stages of PAH.

Since in this study, active RIP3 did not proceed to necroptosis execution in the lungs, and since both RIP3 and MLKL has previously been indicated to mediate activation of NLRP3 inflammasome independently of necroptotic cell death,^13^, ^14^, ^15^ we sought to identify additional RIP3‐dependent pathways which might underlie this tissue injury due to PAH. In this regard, we focused on pyroptosis. In the lungs with PAH, we found upregulated mRNA expression of NLRP3 and elevated protein levels of cleaved csp‐1, which is in accordance with previous studies.^24^, ^60^, ^61^ In line, there was a higher relative processing of IL‐1β and importantly, we also found increased levels of pyroptotic executive protein, N‐terminal GSDMD. According to these findings, it can be postulated that in the lungs, increased phosphorylation of RIP3 might result in the inflammasome formation and pyroptosis execution rather than in the activation of necroptotic cell death. In line, it seems that the upregulation of pulmonary HMGB1 was the result of pyroptotic rather than necroptotic cell death. This observation is consistent with findings of another study showing the elevation of HMGB1 along with activated pyroptosis in the lungs with PAH as well.^24^

We next investigated other inflammasome components. In the lungs, the mRNA expression of NLRC4 was increased in PAH groups while AIM2 protein levels were downregulated in both such diseased groups. Because it is known that the PAH‐affected lungs are characterized by the infiltration of several types of immune cells which express the inflammasomes,^35^ we searched for the potential source of the upregulated inflammasome components, mainly NLRP3 and NLRC4 in the lung tissue. In our hands, the levels of Iba1, a specific marker of monocytes/macrophages activation, were unaltered in the lungs. Thereby, in this case, unlike in other studies,^61^, ^62^ a role of other cells of the innate immune system, such as granulocytes rather than monocytes/macrophages, can be suggested. It should be, however, mentioned that the inflammasomes can also be expressed by various types of non‐immune cells besides immune cells^63^ and thus their role cannot be ruled out either.

In the present study, the potential contribution of NLRP3 inflammasome and pyroptosis with respect to RV damage in PAH was also examined for the first time. The expression of both csp‐1 and IL‐1β was found to be unchanged in both stages of PAH despite the upregulation of their proforms. Interestingly, the levels of N‐terminal GSDMD were even reduced due to PAH. Although there is no literary confrontation for such decrease in N‐terminal GSDMD, these data strongly indicate that the RV of subjects with PAH is unlikely affected by pyroptotic cell death. In addition to NLRP3 inflammasome, we also examined whether some other inflammasome sensors associated with chronic cardiac injury^33^, ^34^ might underlie RV damage due to PAH. Both NLRC4 and AIM2 levels were upregulated in the RVs of both PAH groups. However, because Iba1 expression was comparable in the diseased and non‐diseased groups, it seems that such an increase in the inflammasome sensors is not of the origin of monocytes/macrophages.

**FIGURE 7:**
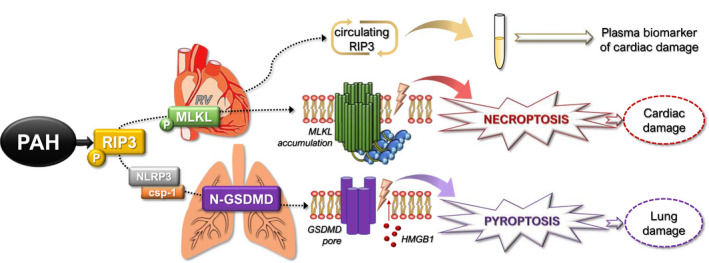
Schematic illustration of the proposed mechanisms of tissue damage under conditions of PAH based on the main findings from this study. In PAH, pThr^231^/Ser^232^‐RIP3 is increased in both RV and lung tissue. In the RVs, such elevation proceeds to the phosphorylation of MLKL, strongly indicating necroptotic cardiac damage, which is likely mediated by the accumulation of MLKL in the plasma membrane causing its rupture. On the contrary, elevated pThr^231^/Ser^232^‐RIP3 in the lungs activates the NLRP3–csp‐1–GSDMD pathway, suggesting pyroptotic damage of the lungs, likely mediated by the creation of GSDMD pores in the plasma membrane resulting in its rupture and release of HMGB1. Furthermore, plasma levels of RIP3 are elevated in PAH and might serve as a potential diagnostic and prognostic biomarker for cardiac injury. (PAH—pulmonary arterial hypertension; RIP3—receptor‐interacting protein kinase 3; RV—right ventricle; MLKL—mixed lineage kinase domain‐like protein; NLRP3—NLR family pyrin domain containing 3; csp‐1—caspase‐1; GSDMD—gasdermin D; HMGB1—high mobility group box 1 protein)

In summary, we showed for the first time that the two most affected tissues by PAH are characterized by the activation of different programmed necrosis‐like cell death forms mediated by pThr^231^/Ser^232^‐RIP3. In the RV, elevation of both pThr^231^/Ser^232^‐RIP3 and pSer^345^‐MLKL was found, thereby clearly indicating the promotion of pro‐necroptotic environment; however in the lung tissue, pThr^231^/Ser^232^‐RIP3 likely proceeded to pyroptotic rather than necroptotic cell death activation (Figure 7). On the contrary, other inflammasome sensors, such as NLRP3 and AIM2 were found to be upregulated in the RV, unlike in the lungs, of animals with PAH. Although we did not examine haemodynamic alterations, which are, however, known from our previous reports,^64^, ^65^ this study also demonstrated that the plasma RIP3 levels are increased in the animals with PAH and are associated with the extent of RV hypertrophy, but not with the markers of haemodynamic stress. Thus, plasma RIP3 might serve as an additional marker of cardiac hypertrophy and myocardial injury due to PAH. Taken together, these findings suggest important and complex role of RIP3 in the pathogenesis of PAH and thus pharmacological targeting of this kinase might be a promising way to prevent cell death in PAH‐induced injury of various tissues with resultant reduction of mortality.

## CONFLICTS OF INTEREST

The authors confirm that there are no conflicts of interest.

## AUTHOR CONTRIBUTION

**Izabela Jarabicova:** Data curation (equal); Formal analysis (equal); Investigation (equal); Methodology (equal); Software (equal); Validation (equal); Writing – original draft (equal); Writing – review & editing (equal). **Csaba Horvath:** Data curation (equal); Formal analysis (equal); Methodology (equal); Software (equal); Visualization (equal); Writing – original draft (equal); Writing – review & editing (equal). **Eva Velasova:** Investigation (supporting); Methodology (supporting); Writing – original draft (supporting). **Lenka Bies Pivackova:** Data curation (supporting); Investigation (supporting); Methodology (supporting); Writing – original draft (supporting); Writing – review & editing (supporting). **Jana Veteskova:** Formal analysis (supporting); Investigation (supporting); Writing – review & editing (supporting). **Jan Klimas:** Funding acquisition (supporting); Methodology (supporting); Resources (supporting); Writing – review & editing (equal). **Peter Krenek:** Data curation (supporting); Funding acquisition (supporting); Methodology (supporting); Resources (supporting); Supervision (supporting); Writing – original draft (supporting); Writing – review & editing (supporting). **Adriana Adameová:** Conceptualization (lead); Data curation (equal); Formal analysis (supporting); Funding acquisition (lead); Investigation (supporting); Methodology (supporting); Project administration (lead); Resources (equal); Software (lead); Supervision (lead); Validation (equal); Writing – original draft (lead); Writing – review & editing (lead).

## Supporting information

Supplementary Material

## Data Availability

Data available on request from the authors
