# Supplementary material for: Analysis of necroptosis and its association with pyroptosis in organ damage in experimental pulmonary arterial hypertension
Source: J Cell Mol Med. 2022 Apr 7;26(9):2633–45. doi: 10.1111/jcmm.17272 (PMC9077306; doi:10.1111/jcmm.17272)
Supplement: Supplementary file 1 — Supplementary Material [file JCMM-26-2633-s001.docx]

**Supplementary material for „Analysis of necroptosis and its association with pyroptosis in organ damage in experimental pulmonary arterial hypertension“**

**Supplementary table 1. Sequences of primers used for RT-qPCR.**

| **Gene** | **RefSeq. Accession No.** | **Sequence (5’-to 3’)** | **Product length (bp)** |
| --- | --- | --- | --- |
| **Hprt1** | NM_012583.2 | Forward: CAGCTTCCTCCTCAGACCG  Reverse: TCACTAATCACGACGCTGG | 82 |
| **Nppb** | NM_031545.1 | Forward: GGAGTCTGCAGCCAGGAGG  Reverse: GACCGGATCGGCGCAGTCA | 78 |
| **Nlrc4** | NM_001309432.1 | Forward: ACTGGAGGCTCAGAGACGAA  Reverse: ATCCATCACTGCTCACACCG | 114 |
| **Nlrp3** | NM_001191642.1 | Forward: AGCCAGAGTGGAATGATGCA  Reverse: CAAATCGAGATGCGGGAGAGA | 112 |

*Abbreviations: Hprt1: hypoxanthine phosphoribosyl-transferase 1; Nppb: brain natriuretic peptide; Nlrc4: NLR family, CARD domain containing 4; Nlrp3: NLR family, pyrin domain containing 3.*

**Supplementary table 2. Average bodyweight of animals, absolute and relative weight of isolated right ventricle**

|  | Control | MCT | ptMCT |
| --- | --- | --- | --- |
| Body weight (g) | 384 ± 7 | 319 ± 8 * | 308 ± 8 * |
| Right ventricle weight (g) | 0,24 ± 0,01 | 0,35 ± 0,03 * | 0,44 ± 0,02 * $ |
| RV w./body w. (mg/g) | 0,61 ± 0,01 | 1,10 ± 0,10 * | 1,44 ± 0,06 * $ |

*Data are presented as mean ± SEM; n = 10 per group; MCT – monocrotaline group; ptMCT – prematurely sacrificed monocrotaline group; * P < 0.05 vs. Control; $ P < 0.05 vs. MCT*

**Supplementary table 3. Hemoglobin oxygen saturation, heart and breath rate of animals measured under passivity and activity conditions**

|  | passive conditions | | | active conditions | | |
| --- | --- | --- | --- | --- | --- | --- |
|  | **Control** | **MCT** | **ptMCT** | **Control** | **MCT** | **ptMCT** |
| oxygen saturation (%) | 94,1 ± 0,2 | 91,6 ± 0,9 | 92,0 ± 0,7 | 93,7 ± 0,2 | 91,3 ± 0,9 | 91,9 ± 0,6 |
| heart rate (min^-1^) | 396,9 ± 7,3 | 458,4 ± 11,1* | 451,2 ± 8,3* | 432,5 ± 5,1 | 468,4 ± 10,2* | 449,9 ± 8,0 |
| respiratory rate (min^-1^) | 103,6 ± 2,7 | 121,1 ± 7,0 | 156,7 ± 9,3*$ | 100,5 ± 1,5 | 117,3 ± 5,4 | 147,7 ± 9,5*$ |

*Data are presented as mean ± SEM; n = 10 per group; MCT – monocrotaline group; ptMCT – prematurely sacrificed monocrotaline group; * P < 0.05 vs. Control; $ P < 0.05 vs. MCT*

**Supplementary figure 1:** Correlation analysis between the main necroptotic proteins in the RV and markers of cardiac injury. Correlation between RIP3 and Nppb in the RV (A), the plasma NT-proBNP (B), Fulton index (C); between pThr^231^/Ser^232^-RIP3 and Nppb in the RV (D), the plasma NT-proBNP (E), Fulton index (F); between pSer^345^-MLKL and Nppb in the RV (G), the plasma NT-proBNP (H), Fulton index (I) in monocrotaline group (MCT) and prematurely terminated monocrotaline group (ptMCT); n = 9-10 per group; n.s. – non-significant

**Supplementary figure 2:** Correlation analysis between the plasma levels of RIP3 and vital functions. Correlation between the plasma RIP3 and breath rate under passive conditions (A), breath rate under active conditions (B), heart rate under passive conditions (C), heart rate under active conditions (D) in monocrotaline group (MCT) and prematurely terminated monocrotaline group (ptMCT); n = 5-9 per group; n.s. – non-significant
